# Allele Frequencies of Genetic Variants Associated with Varroa Drone Brood Resistance (DBR) in *Apis mellifera* Subspecies across the European Continent

**DOI:** 10.3390/insects15060419

**Published:** 2024-06-04

**Authors:** Regis Lefebre, Lina De Smet, Anja Tehel, Robert J. Paxton, Emma Bossuyt, Wim Verbeke, Coby van Dooremalen, Zeynep N. Ulgezen, Trudy van den Bosch, Famke Schaafsma, Dirk-Jan Valkenburg, Raffaele Dall’Olio, Cedric Alaux, Daniel S. Dezmirean, Alexandru I. Giurgiu, Nuno Capela, Sandra Simões, José Paulo Sousa, Martin Bencsik, Adam McVeigh, Michael Thomas Ramsey, Sausan Ahmad, Tarun Kumar, Marc O. Schäfer, Alexis L. Beaurepaire, Arrigo Moro, Claude J. Flener, Severine Matthijs, Dirk C. de Graaf

**Affiliations:** 1 Department of Biochemistry and Microbiology, Ghent University, 9000 Ghent, Belgium; lina.desmet@ugent.be (L.D.S.); embossuy.bossuyt@ugent.be (E.B.); dirk.degraaf@ugent.be (D.C.d.G.); 2 Institute for Biology, Martin Luther University Halle-Wittenberg, D-06126 Halle (Saale), Germany; anja.tehel@zoologie.uni-halle.de (A.T.); robert.paxton@zoologie.uni-halle.de (R.J.P.); 3 Department of Agricultural Economics, Ghent University, 9000 Ghent, Belgium; wim.verbeke@ugent.be; 4 Wageningen Plant Research, Wageningen University & Research, 6708 PB Wageningen, The Netherlandszeynep1.ulgezen@wur.nl (Z.N.U.); trudy.vandenbosch@wur.nl (T.v.d.B.); famke.schaafsma-terburg@wur.nl (F.S.); dirkjan.valkenburg@wur.nl (D.-J.V.); 5 BeeSources, 40132 Bologna, Italy; raffaele.dallolio@gmail.com; 6 Unité Abeilles et Environnement, Institut National de la Recherche pour l’Agriculture, CEDEX 9, 84914 Avignon, France; cedric.alaux@inrae.fr; 7 Apiculture and Sericulture Unit, University of Agricultural Sciences and Veterinary Medicine of Cluj-Napoca, 400372 Cluj Napoca, Romania; ddezmirean@usamvcluj.ro (D.S.D.); alexandru.giurgiu@usamvcluj.ro (A.I.G.); 8 Centre for Functional Ecology, Associate Laboratory TERRA, Department of Life Sciences, University of Coimbra, 3000-456 Coimbra, Portugal; nuno.capela@uc.pt (N.C.); sandra.simoes@uc.pt (S.S.); jps@zoo.uc.pt (J.P.S.); 9 Physics and Mathematics, Nottingham Trent University, Nottingham NG8 11NS, UK; martin.bencsik@ntu.ac.uk (M.B.); adam.mcveigh@murdoch.edu.au (A.M.); michaelthomas.ramsey@outlook.com (M.T.R.); sony76@hotmail.co.uk (S.A.); kumart1@aston.ac.uk (T.K.); 10 Institute of Infectology, Federal Research Institute for Animal Health, Friedrich-Loeffler-Institut, 17493 Greifswald-Insel Riems, Germany; marc.schaefer@fli.de; 11 Institute of Bee Health, University of Bern, 3003 Bern, Switzerland; alexis.beaurepaire@unibe.ch (A.L.B.); arrigo.moro@universityofgalway.ie (A.M.); 12 Center for Bee Research, 3097 Liebefeld, Switzerland; 13 Galway Honey Bee Research Centre, Department of Zoology, University of Galway, H91 TK33 Galway, Ireland; 14 Finnish Beekeeper’s Association (SML), 00130 Helsinki, Finland; claude@aitonatura.com; 15 Department of Viral Reemerging, Enzootic and Bee Diseases, Sciensano, 1050 Elsene, Belgium; severine.matthijs@sciensano.be

**Keywords:** western honey bee, marker-assisted selection, genetic markers, suppressed mite reproduction, varroa mite non-reproduction

## Abstract

**Simple Summary:**

Increasing honey bee resilience against the parasitic mite *Varroa destructor*, while respecting sustainability, may be achieved by enriching natural mite resistance traits in the breeding population. Some of these traits are linked to specific variants in the honey bee genome, which can be pinpointed and characterized with high-throughput molecular lab tests. Breeding by focusing on these genomic variants is thus more efficient and less time-consuming. However, when evaluating the link between a specific varroa resistance trait outcome and its associated variants in the genome, different contributions of the variants may be observed between honey bee races. It is hypothesized that these observations evolve from different linkages between the trait-causing genes and the genomic variants. Therefore, we evaluated the presence of genomic variants associated with a varroa resistance trait in different honey bee samples across the European continent. We observed significant differences in the presence of the genomic variants in the considered honey bee races, which underpin our hypothesis of linkage dissimilarities. This study shows that determining the honey bee race prior to using the genomic variants associated with varroa resistance for selective breeding is of utmost importance.

**Abstract:**

Implementation of marker-assisted selection (MAS) in modern beekeeping would improve sustainability, especially in breeding programs aiming for resilience against the parasitic mite *Varroa destructor*. Selecting honey bee colonies for natural resistance traits, such as brood-intrinsic suppression of varroa mite reproduction, reduces the use of chemical acaricides while respecting local adaptation. In 2019, eight genomic variants associated with varroa non-reproduction in drone brood were discovered in a single colony from the Amsterdam Water Dune population in the Netherlands. Recently, a new study tested the applicability of these eight genetic variants for the same phenotype on a population-wide scale in Flanders, Belgium. As the properties of some variants varied between the two studies, one hypothesized that the difference in genetic ancestry of the sampled colonies may underly these contribution shifts. In order to frame this, we determined the allele frequencies of the eight genetic variants in more than 360 *Apis mellifera* colonies across the European continent and found that variant type allele frequencies of these variants are primarily related to the *A. mellifera* subspecies or phylogenetic honey bee lineage. Our results confirm that population-specific genetic markers should always be evaluated in a new population prior to using them in MAS programs.

## 1. Introduction

Due to a limited interspecific co-evolution between the varroa mite (*Varroa destructor)* and its novel host, the western honey bee (*Apis mellifera*), the ectoparasitic mite is repeatedly attributed as a main driver of *A. mellifera* colony losses by affecting the bees’ body weight, immune system, metabolism, homing abilities and reproductive capacities and acting as a vector of virulent viruses like the deformed wing virus [1,2,3,4,5,6,7,8,9,10,11,12,13,14,15]. Most beekeepers apply chemical acaricide treatments to control the mite infestations in their colonies, but these methods are unsustainable as they contaminate many bee products and have led to reduced efficacy and resistance in mites [16,17,18,19].

The goal of sustainable beekeeping is the long-term maintenance of honey bee colonies with respect to their natural behavior and local adaptation. One sustainable way of applying varroa mite control is through the selective breeding of colonies expressing naturally occurring, heritable mite resistance traits [20,21,22,23]. For example, Varroa Sensitive Hygiene, the detection and removal of varroa-infested brood, mainly explained mite resistance in naturally surviving colonies from Avignon, France [21,24]. Other colonies have shown to express suppression of mite reproduction (SMR), a colony-level trait in which brood-invading varroa foundress mites are unable to start or complete their reproductive cycle [21,24,25].

Colony-level SMR is the holistic summation of trait outcomes such as from the earlier mentioned Varroa Sensitive Hygiene, in which hygienic bees would prefer to remove pupae with reproducing mites [26,27,28,29], or brood-intrinsic resistance traits deriving from disturbances in the close host-parasite physiological interactions [30,31,32]. The interplay of these traits has been reviewed in [33]. Brood-intrinsic suppression of mite reproduction may be expressed in two different ways [34]. The first is a fecundity-based reduction in mite reproduction, which is characterized by the production of a lower number of viable and/or mated daughter mites by the mother mite compared to commonly reported maxima [34]. The second, mite non-reproduction (MNR), is defined as the brood-intrinsic trait wherein foundress mites are incapable of producing any offspring, either male or female. This trait is also referred to as fertility-based reduction in mite reproduction [34]. If MNR is expressed in drone or worker brood, one refers to it as drone brood resistance (DBR) and worker brood resistance (WBR), respectively [35].

However, phenotypic scores on these mite resistance traits are often unprecise, unrepeatable and labor-intensive to evaluate [29,34]. Shifting from phenotyping to genotyping through the implementation of marker-assisted selection (MAS) may be more efficient in integrating these traits in honey bee breeding programs. MAS involves the identification of specific genetic markers associated with a trait of interest, which can then be used as molecular indicators in selection processes. These markers are genome variations that are linked to genes controlling the trait of interest, like disease resistance, crop yield, quality or tolerance to environmental stresses. The simplest forms of genetic markers are single nucleotide polymorphisms (SNPs), which are variations of only one nucleotide at specific loci in the genome.

Broeckx et al. (2019) identified eight SNPs in the honey bee genome associated with DBR after phenotyping this trait in capped drone brood from an artificially created hybrid varroa-resistant/varroa-sensitive queen [35]. This hybrid queen, descending from the Amsterdam Water Dune population in the Netherlands, was the only queen showing a significantly higher percentage of DBR compared to a local control colony [35]. Subsequently, this queen’s phenotyped drone pupae were used for genetic marker identification, resulting in an eight-variant model that classified 88% of the drones’ DBR phenotypes correctly [35]. The model comprised six risk SNPs and two protective SNPs, depending on whether the variant type (Vt) allele for the SNP increased or decreased the probability of reproduction of the included varroa mite, respectively. It is important to note that these eight DBR-associated variants descended from only one hybrid colony and that it was unknown whether these markers could be used for marker-assisted selection on a population-wide scale, including different genetics and beekeeping practices, or even in a different *A. mellifera* subspecies.

Recently, the above-mentioned eight variants were evaluated for their applicability on a population-wide scale in Flanders, Belgium, by sampling more than 160 different *A. mellifera carnica* colonies [36]. Notably, the researchers in this study built a reduced three-variant model retaining only three of the eight genetic variants and found that their model classified 76% of the 842 DBR-phenotyped drones correctly. However, two out of three remaining variants switched properties in the reduced model when compared to the original eight-variant model [35,36]. One SNP changed from being a risk mutation in the original model to being a protective mutation in the new reduced model (SNP2), while another switched from being originally protective to being a risk variant (SNP6) [36]. One possible explanation for this could be the difference in genetic ancestry of the sampled honey bee colonies in both studies. As genetic markers are linked to loci in genes that control the trait of interest (here: DBR), this marker–loci linkage may differ between the *A. mellifera* subspecies from which the models were constructed [36,37]. However, the DBR-related loci are still unknown, and identifying them would enable us to test the linkage dissimilarity hypothesis stated here.

The original eight-variant model was derived from a colony that evolved from the Darwinian Black Bee Box (DBBB) program, wherein managed honey bee colonies are subject to natural selection by stopping mite treatment, allowing varroa resistance to establish [38]. Moreover, mating is only allowed within the (isolated) DBBB population, and selection is based on the proliferation of mite-surviving colonies. This strategic breeding program specifically focuses on the conservation of the European black bee or *A. m. mellifera*, whereas the sampled subspecies in the Flemish population-wide validation study was the Carniolan honey bee or *A. mellifera carnica*, preferred by Flemish beekeepers due to its gentleness and productivity [36]. As *A. mellifera carnica* belongs to the evolutionary *A. mellifera* C lineage [39], the researchers hypothesized that the reported three variant model, and thus the properties of the three variants, could potentially be applied in breeding programs considering other subspecies belonging to the same lineage [36,40,41]. Breeding programs focusing on subspecies from, for example, the A or M lineage, could probably not profit from this reduced three-variant model [36]. Examples of non-C lineage subspecies in Europe are *A. mellifera iberiensis* and *A. m. mellifera* [41].

In this study, the Vt allele frequencies of all eight genetic markers associated with DBR in the single Amsterdam Water Dune colony were determined in more than 360 pooled worker bee samples from different honey bee colonies across the European continent. Moreover, for some colonies, the *A. mellifera* subspecies was determined using the SMART bees SNP chip, and correlations of specific subspecies and phylogenetic lineages with Vt allele frequencies were analyzed. In this way, the present study functions as an explorative screening for the eight relevant SNPs in different countries and subspecies on the European Continent, and may pave the way for country- and/or subspecies-specific population-wide genotype–phenotype association studies, similar to the one reported for *A. mellifera carnica* colonies in Flanders, Belgium [36].

## 2. Materials and Methods

### 2.1. Sampling and Sample Distribution

**Figure 1:**
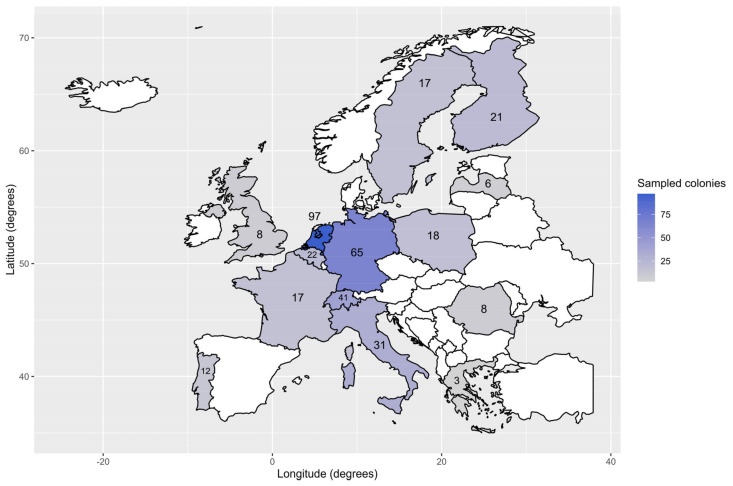
Number of sampled colonies per European country involved in the study. Non-participating countries are coloured white. The colour legend (right) gives an indication of the number of sampled colonies. Most samples came from the Netherlands (*n* = 97), Germany (*n* = 65) and Switzerland (*n* = 41).

In the frame of the EU-funded B-GOOD project, a total of 366 *A. mellifera* colonies across 14 European countries were sampled for adult worker bees during the autumn of 2022 (Figure 1, Supplementary Table S1). Participating countries were Belgium (BE), Finland (FI), France (FR), Germany (DE), Greece (GR), Italy (IT), Latvia (LV), Poland (PL), Portugal (PT), Romania (RO), Sweden (SE), Switzerland (CH), the Netherlands (NL) and the United Kingdom (UK). The sampling strategy comprised countries located along the longitudinal axes of the continent as well as countries spread along its latitudinal axes. However, the number of sampled colonies was not equally distributed among the countries. For example, most colonies were sampled in The Netherlands (*n* = 97), followed by Germany (*n* = 65) and Switzerland (*n* = 41). The lowest numbers of colonies were sampled in Greece, Latvia, Romania and the United Kingdom (*n* = 3, 6, 8 and 8, respectively).

From each colony involved in the study, 15 *A. mellifera* worker bees were pooled in vials during the autumn of 2022. Samples from Italy, France, Finland, Greece, the United Kingdom, Portugal and Belgium were sent to Sciensano (SCIEN, Belgium) for subsequent gDNA extraction, while samples from Germany, Latvia, Poland, Romania, Sweden, Switzerland and the Netherlands were sent to the Friedrich-Loeffler-Institut (FLI, Germany) for subsequent gDNA extraction. All samples were chilled during handling and transported on dry ice.

### 2.2. Genomic DNA Extractions from Pooled Worker Bee Samples

Protocols for gDNA extraction from the pooled worker bees differed between SCIEN and FLI. In both laboratories, 15 bees per sample were homogenized in 7.5 mL PBS (pH 7.4) using a gentleMACS^TM^ Dissociator (Miltenyi Biotec) at FLI or a TissueLyser (Qiagen) at SCIEN. Afterwards, the homogenates were conserved in ultrafreezers (−80 °C) until nucleotide extraction. DNA was extracted from the clarified homogenates using the standard protocol of the NucleoMag VET-kit (FLI) or the standard protocol of the IndiMag Pathogen kit (Indical Bioscience) (SCIEN). Both protocols involve rapid and automated adsorption and purification of nucleic acids to paramagnetic beads, including a lysis step, binding step, multiple washing steps and a final elution step.

### 2.3. Single Thorax gDNA Extractions from Local Apiary Bees

Worker bees were sampled randomly from Belgian colonies and genomic DNA was extracted individually to genotype for the eight genetic variants associated with DBR using qPCR assays with dual-labelled probes [42] (cf. 2.4. qPCR assays with dual-labelled probes). For each individual worker bee, the thorax was dissected and homogenized with 0.5 mL lysis buffer (100 mM NaCl; 20 mM Tris-HCl, pH 8; 25 mM EDTA, pH 8; 0,5% SDS) and metal and zirconium beads for 1 min at 30 Hz (PowerLyzer^®^ 24 Homogenizer, Qiagen). After incubation with 10 µL proteinase K (20 mg/mL) at 56 °C for 4 h, an equal volume of phenol:chloroform:isoamylalcohol was added, vortexed and centrifuged at 12,000× *g* for 30 min at 4 °C, followed by transfer and extraction of the supernatant with an equal volume of chloroform and centrifugation at 12,000× *g* for 15 min at 4 °C. Next, gDNA was precipitated from the supernatant by addition of two volumes of ice cold 100% ethanol and overnight incubation at −20 °C. After centrifugation for 30 min at 12,000× *g*, the DNA pellet was washed with 70% ethanol, air-dried and resuspended in 100 µL DNase/RNase-free water. DNA concentrations were measured by spectrophotometry.

### 2.4. qPCR Assays with Dual-Labelled Probes

For each gDNA sample to be analysed, eight distinct genotyping assays were performed in a 10 µL reaction volume containing 1× KEY buffer, 250 nM of each primer, 250 nM of each dual-labelled probe, 200 µM of each dNTP, 0.5 U TEMPase Hot Start DNA Polymerase (VWR) and 20 ng gDNA [42]. qPCR assays were run on the Bio-Rad C1000TM Thermal Cycler with CFX96TM Real-Time System with one cycle of 95 °C for 14 min 40 s, followed by 60 × (95 °C for 20 s, followed by 40 s of the assay-specific annealing/elongation/signal detection temperature [42]). Data analysis was conducted using the Bio-Rad CFX Manager 3.1 Software.

### 2.5. Calibration Curve Construction and Allele Frequency Analysis in Pooled Worker Bee Samples

All individual worker bee gDNA samples from the sampled Belgian *A. mellifera* colonies were genotyped for the eight genetic variants using qPCR assays with dual-labelled probes. One homozygous wild type and one homozygous variant type bee were selected per genetic marker. Each selected gDNA sample was diluted to 10 ng/µL and calibration curves were constructed by pooling proportions of volumes of wild type (Wt) and variant type (Vt) gDNA equal to the percentages of Wt and Vt alleles in the calibration curve samples. In each qPCR assay with dual-labelled probes for variant type allele frequency analysis in the European pooled bee samples, calibration curve standards of 0%, 20%, 40%, 50%, 60%, 80% and 100% Vt allele were run in duplicate. For each genetic variant, the percentage of the Vt allele in a pooled worker bee gDNA sample was determined by retrograde regression analysis of the end-RFU value of the Vt allele’s fluorophore in the sample vs. the biquadratic intraplate calibration curve.

### 2.6. SMART Bees SNP Chip Analysis

For subspecies determination, DNA was extracted from one worker bee per colony (subset; *n* = 139) and commercially genotyped at 4094 SNPs selected to provide an accurate prediction of subspecies ancestry (Eurofins Genomics) [43]. Subspecies assignment was done by machine learning (linear support vector classification; [43]) using a reference data set of 14 subspecies of *A. mellifera* (ssp. *adami*, ssp. *anatoliaca*, ssp. *armeniaca*, ssp. *carnica*, ssp. *carpatica*, ssp. *caucasica*, ssp. *cecropia*, ssp. *cypria*, ssp. *iberiensis*, ssp. *ligustica*, ssp. *macedonica*, ssp. *mellifera*, ssp. *rodopica* and ssp. *ruttneri*). Subspecies genotype was assigned based on the highest probability of group membership.

### 2.7. Statistics

Plots, graphs, maps and statistics were constructed/performed using R Statistical Software (v4.2.2; R Core Team 2021) [44]. All tests were checked for and complied with the required assumptions and evaluated at the (Bonferroni-corrected) 5% significance level (α = 0.05). First, variant type allele frequencies in pooled worker bee samples (one pool per colony) were grouped per country, and differences in allele frequency distributions between countries were tested with the Kruskal–Wallis Test [45] and post-hoc Bonferroni-corrected pairwise Dunn’s Tests [46,47]. Next, the percentages of variant type alleles in pooled worker bee samples were plotted in relation to the subspecies of the colonies from which the samples were taken, and differences in allele frequency distributions between subspecies at the European and/or country level were tested similarly (Kruskal–Wallis Test and post-hoc Bonferroni-corrected pairwise Dunn’s Tests).

For logistic regression modelling, specific subspecies or genetic lineages were dummy-coded first. Logistic regression models with percentages of variant type alleles for all eight SNPs as predictors and the dummy-coded output variable were built with SPSS Statistics 29. To evaluate the relevance of the predictors, null models were compared with predictor-containing models via Omnibus Tests, with the significance level set at 5%. The Negative Predictive Value (NPV), Positive Predictive Value (PPV), sensitivity and specificity of each model were calculated based on the model’s classification table.

## 3. Results

### 3.1. Country-Specific Distributions of Variant Type Allele Frequencies of the Eight Genetic Variants Associated with DBR

**Figure 2:**
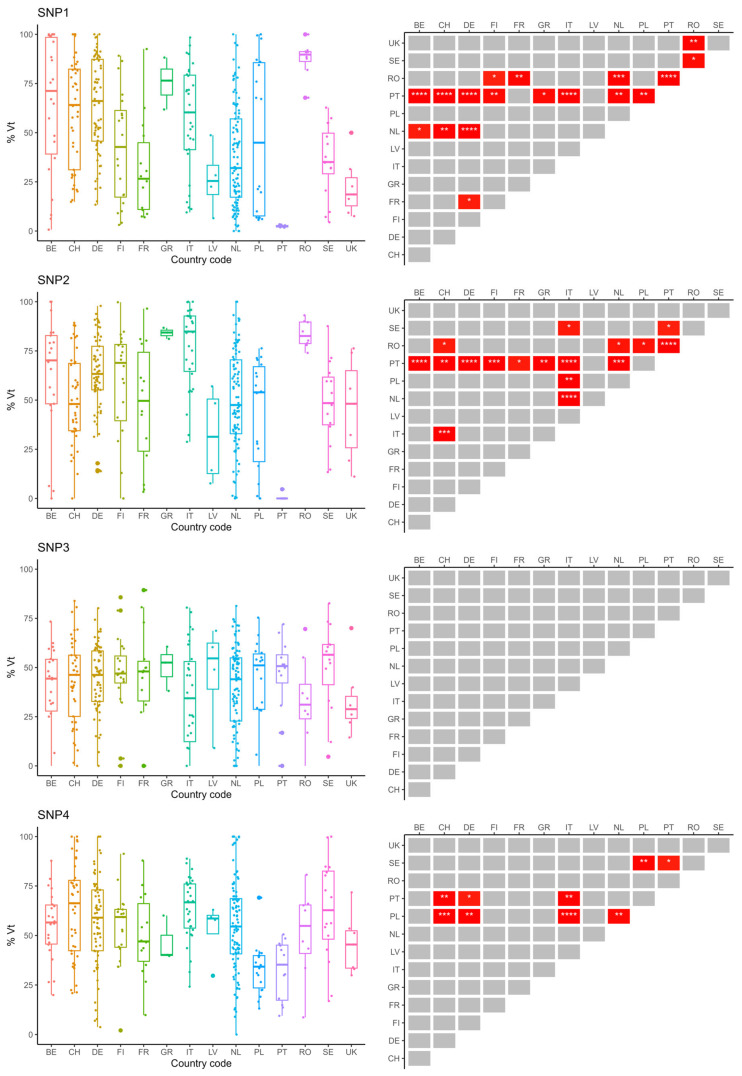

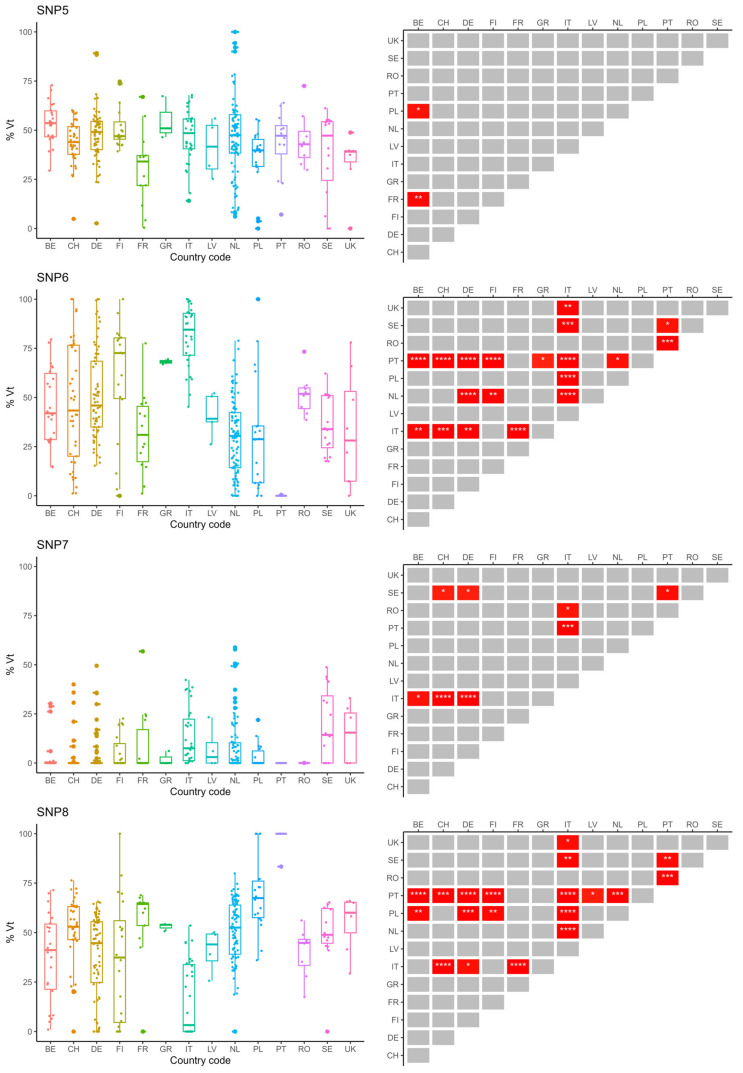
Variant type (Vt) allele frequency distributions of the eight DBR-associated genetic variants in pooled worker bee samples from colonies across the European continent. SNP numbers are designated as in Supplementary Table S2 and [42]. Boxplots (**left**) and Dunn’s Tests pairwise significance matrix (**right**) are shown per SNP. Detailed information (means, medians, variance,…) for the variant type allele frequency distributions can be found in Supplementary Table S3. Country codes: BE = Belgium; CH = Switzerland; DE = Germany; FI = Finland; FR = France; GR = Greece; IT = Italy; LV = Latvia; NL = the Netherlands; PL = Poland; PT = Portugal; RO = Romania; SE = Sweden; UK = United Kingdom. In the significance matrices, asterisks indicate Bonferroni-corrected significance levels *p* ≤ 0.05 (*), *p* ≤ 0.01 (**), *p* ≤ 0.001 (***) or *p* ≤ 0.0001 (****). Grey blocks indicate non-significant pairwise differences after Bonferroni correction. The Bonferroni-corrected significance level α was set at 0.05.

Differences in allele frequency distributions between countries were found for all SNPs, except SNP3 (Kruskal–Wallis H = 13.6, df = 13; *p* = 0.405) (Figure 2, Supplementary Table S3). The variant type allele frequencies for SNP3 had similar distributions in all sampled countries.

Most differences in allele frequency distributions between countries were found for SNP1 (N_sign_ = 18), SNP2 (N_sign_ = 17), SNP6 (N_sign_ = 19) and SNP8 (N_sign_ = 19), indicating high variability and location-dependency of frequencies of the variant type allele for these SNPs. For instance, for SNP1 and SNP2, the variant type allele frequencies in samples from Portugal and Romania differed significantly from those in numerous other countries, showing lower and higher percentages than the others, respectively. For SNP6 and SNP8, the same held true when considering samples from Portugal and Italy. Portugal and Poland had lower percentages of variant type alleles in pooled worker bee samples for SNP4 compared to Switzerland, Germany, Italy and Sweden. A limited, though significant (Kruskal–Wallis: *p* = 0.0008) number of differences in the distributions of the variant type allele between countries could be found for SNP5 (PL-BE (*) and FR-BE (**)). The variant type allele frequencies for SNP7 seemed to be low in all sampled countries. However, the samples from Italy and Sweden did show significantly higher variant type allele frequencies for SNP7 when compared to other countries such as Belgium, Switzerland, Germany, Romania and Portugal.

### 3.2. A. mellifera Subspecies Determination with SMART Bees SNP Chip

**Figure 3:**
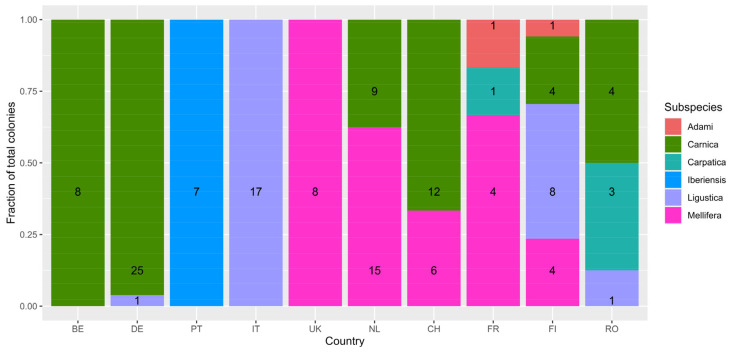
Fractions of detected *A. mellifera* subspecies and total number of genotyped colonies among the sampled countries. Per country, coloured bars indicate the fraction of the total number of genotyped colonies assigned to a specific subspecies. Numbers represent the number of colonies of the detected subspecies. Country codes: BE = Belgium; DE = Germany; PT = Portugal; IT = Italy; UK = United Kingdom; NL = The Netherlands; CH = Switzerland; FR = France; FI = Finland; RO = Romania.

For 139 of the 366 sampled colonies, the *A. mellifera* subspecies was determined using the SMART bees SNP chip (Figure 3). This was only done for a portion of sampled colonies from Belgium (*n* = 8), Germany (*n* = 26), Portugal (*n* = 7), Italy (*n* = 17), United Kingdom (*n* = 8), the Netherlands (*n* = 24), Switzerland (*n* = 18), France (*n* = 6), Finland (*n* = 17) and Romania (*n* = 8). The most common subspecies among all tested colonies was *A. mellifera carnica* (62/139 = 44.6%), mostly found in Belgium, Germany, the Netherlands and Switzerland. *A. mellifera iberiensis* (formerly ssp. *iberica*) was only found in Portugal, where all colonies comprised ssp. *iberiensis*. All tested colonies from Italy were genotyped as *A. mellifera ligustica* and all tested samples from the United Kingdom were genotyped as *A. m. mellifera*. *A. m. mellifera*, or the European dark bee, was also found in the Netherlands, Switzerland, France and Finland. A limited number of colonies were genotyped as ssp. *adami* or ssp. *carpatica*, possibly reflecting the Buckfast commercial hybrid honey bee variant or Balkan honey bees, respectively. The vast majority of tested colonies from Belgium and Germany (DE: 25 of 26) were genotyped as ssp. *carnica*.

### 3.3. Subspecies-Specific Distributions of Variant Type Allele Frequencies of the Eight Genetic Variants Associated with DBR

**Figure 4:**
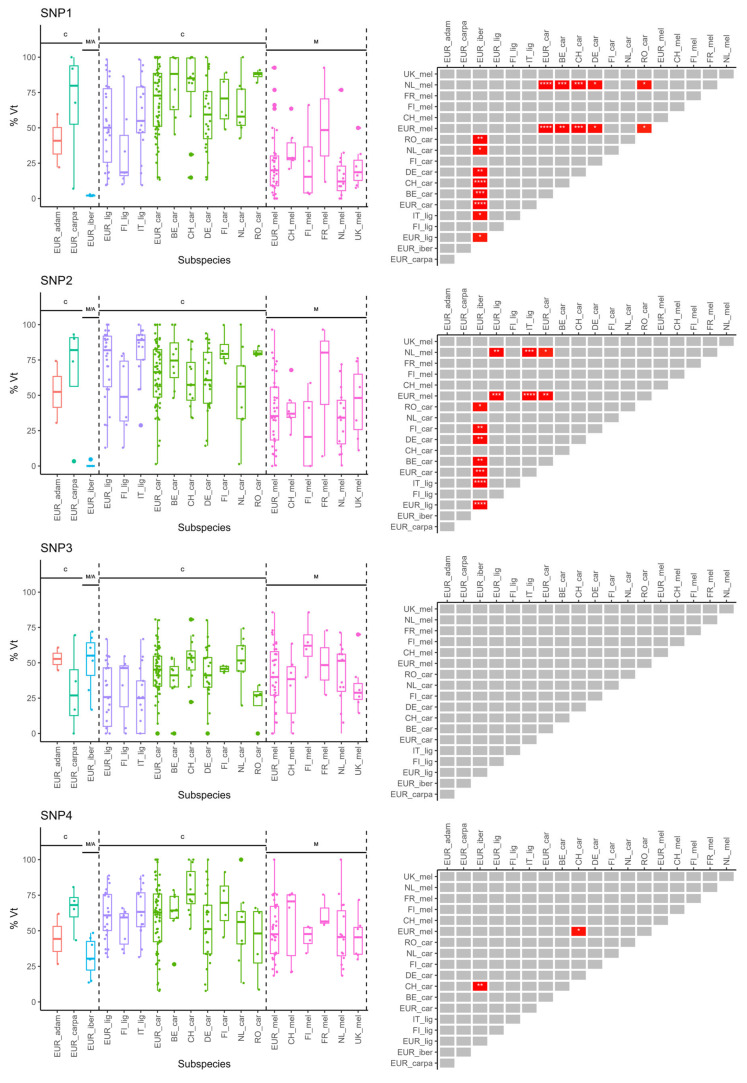

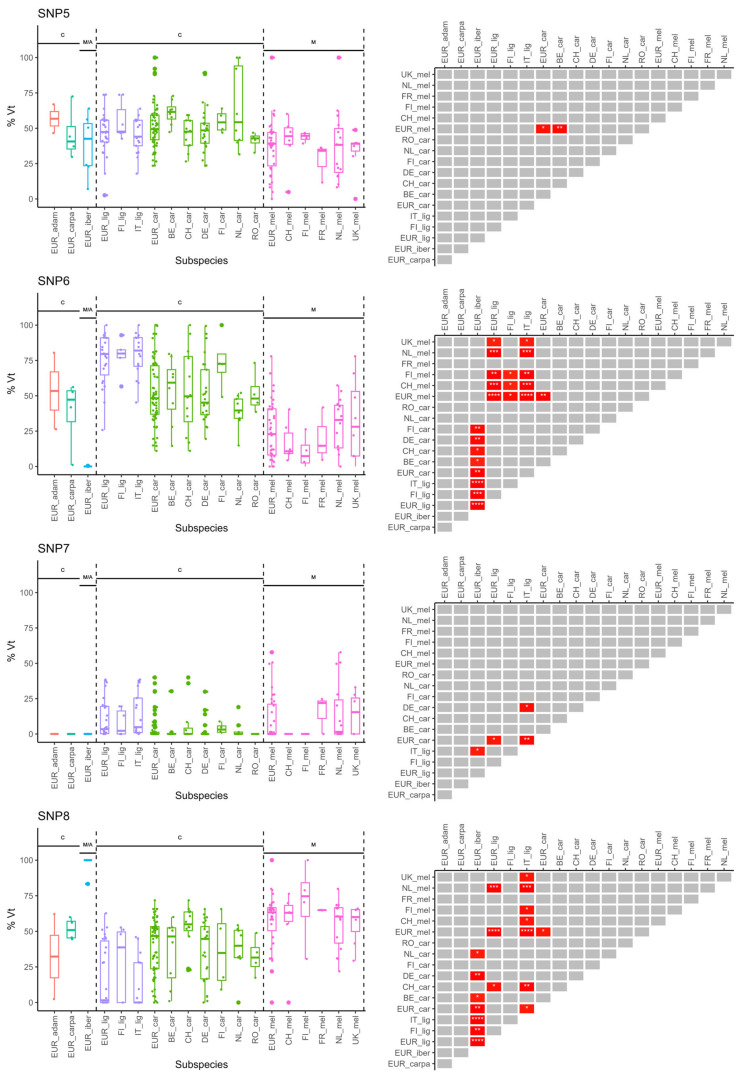
Subspecies-grouped variant type allele frequency distributions of the eight genetic variants associated with DBR. Boxplots (**left**) and Dunn’s Tests pairwise significance matrix (**right**) are shown per SNP. Percentages of variant type alleles are represented in relation to the subspecies at the overall European level (‘EUR_ssp.’; all tested samples of that subspecies) and the subspecies by country (‘Country_ssp.’; for countries with at least 4 samples of the respective subspecies). Country codes: FI = Finland; IT = Italy, BE = Belgium; CH = Switzerland; DE = Germany; NL = the Netherlands; RO = Romania; FR = France; UK = United Kingdom. C: subspecies belonging to the C-lineage; M: subspecies belonging to the M-lineage; M/A: subspecies belonging to M- or A-lineage. Asterisks indicate Bonferroni-corrected significance levels *p* ≤ 0.05 (*), *p* ≤ 0.01 (**), *p* ≤ 0.001 (***) or *p* ≤ 0.0001 (****) in the significance matrices. Grey blocks indicate non-significant pairwise differences after Bonferroni correction. The Bonferroni-corrected significance level α was set at 0.05.

For all eight genetic variants, the percentages of variant type alleles in the pooled worker bee samples were plotted in relation to the subspecies of the colonies from which the samples were taken (*n* = 139; Figure 4, Supplementary Table S4). Per subspecies, this was done at the European level (for example ‘EUR_lig’, i.e., all samples genotyped as ssp. *ligustica* among the European samples) and at the country level if N_ssp._ ≥ 4 (e.g., ‘FI_lig’ = all samples genotyped as ssp. *ligustica* from Finland). This allowed us to detect differences in distributions between samples from colonies of the same subspecies but from different countries.

Again, no differences in allele frequency distributions were found for SNP3 (Figure 4). In other words, the variant type allele frequency of SNP3 in pooled worker bees is uncorrelated with the subspecies or country of sampling. For SNP1 and SNP2, similar patterns in the Dunn’s Tests significance matrices were detected. For example, the percentages of the variant type allele in ssp. *iberiensis* samples (i.e., all samples from Portugal) for both SNP1 and SNP2 differed significantly from those in many ssp. *carnica* and ssp. *ligustica* samples, but not from ssp. *mellifera* samples. Moreover, the overall ssp. *mellifera* samples in Europe and ssp. *mellifera* samples from the Netherlands showed significantly different percentages of the variant type allele when compared to ssp. *carnica* samples and ssp. *ligustica* samples for SNP1 and SNP2 respectively. For these two SNPs, no significant differences in percentages of the variant type allele between samples of the same subspecies, but from different countries, were detected. Percentages of the variant type allele for SNP1 and SNP2 in ssp. *adami* and ssp. *carpatica* samples did not differ from other subspecies; however, it should be noted that for these two subspecies, sample sizes were small (*n* = 2 and *n* = 4 respectively).

Only the percentages of the variant type allele for SNP4 in the ssp. *carnica* samples from Switzerland were significantly higher than those in ssp. *iberiensis* and ssp. *mellifera* samples at the European level (*p* = 0.002** and *p* = 0.02*, respectively). These results suggest a higher distribution of percentages of variant type allele for SNP4 in ssp. *carnica* samples from Switzerland compared to ssp. *carnica* samples from other countries, although this difference was not significant after Bonferroni correction.

Samples from the ssp. *mellifera* genotype at the European level showed slightly lower percentages of the variant type allele for SNP5 when compared to all samples from the ssp. *carnica* genotype (‘EUR_car’). This difference is probably influenced by the ssp. *carnica* samples from Belgium, which showed higher percentages of the variant type allele than samples of the ssp. *mellifera* genotype at the European level too (*p* = 0.009**). Samples of the ssp. *adami*, ssp. *carpatica*, ssp. *iberiensis* and ssp. *ligustica* genotypes did not show significantly different distributions when compared to each other, or to samples of the ssp. *carnica* genotype or the ssp. *mellifera* genotype.

The samples from Portugal (ssp. *iberiensis*) showed significantly lower percentages of the variant type allele for SNP6 when compared to samples of the ssp. *carnica* and the ssp. *ligustica* genotype. Moreover, percentages of the variant type allele for SNP6 were significantly higher in samples of the ssp. *ligustica* genotype than in ssp. *mellifera* samples. *Apis m. mellifera* samples (‘EUR_mel’) also showed a significantly lower distribution in percentages of the variant type allele for this SNP when compared to ssp. *carnica* samples in the overall European group, but it was not significantly different from the ssp. *iberiensis* samples.

Both the boxplots and significance matrix show that the percentages of the variant type allele for SNP7 are slightly higher in ssp. *ligustica* than in ssp. *carnica*, but not when compared to samples of the ssp. *mellifera* genotype. However, ssp. *mellifera* samples did not show significantly higher percentages of the variant type allele for SNP7 when compared to other subspecies.

Comparable differences in the variant type allele frequency distributions were found for SNP8, as was the case for SNP6. More specifically, the samples from Portugal (*iberiensis* ssp.) showed significantly higher (instead of lower for SNP6) percentages of the variant type allele compared to samples from the ssp. *carnica* and ssp. *ligustica* genotypes, but not in comparison with samples of ssp. *mellifera*. Samples of the latter-mentioned genotype did show significantly higher percentages of the variant type allele for SNP8 than samples from the ssp. *ligustica* and ssp. *carnica* genotypes.

### 3.4. Subspecies-Specific Logistic Regression Models

Alternatively, by constructing logistic regression models with a predefined dummy-coded subspecies as the output variable and the percentages of variant type allele for each of the eight SNPs as continuous predictor variables, we could emphasize specific associations between certain SNPs and each of the detected subspecies (Table 1). More specifically, the SNPs that are significant predictors for a dummy-coded subspecies in these models are the SNPs showing significantly differing distributions from other subspecies. A positive estimate for a significant SNP as a predictor indicates a higher probability on the predefined dummy-coded subspecies with increasing percentages of variant type allele for that SNP. For samples of the ssp. *carnica* genotype, the model suggested that higher percentages of the variant type allele for SNP1 are characteristic of this genotype (Estim = +0.059; *p* < 0.001***), a finding that could be validated by the significance matrix of SNP1 (cf. Figure 4). For ssp. *ligustica*, lower percentages of the variant type allele for SNP1 (Estim = −0.043; *p* = 0.04*) and SNP8 (Estim = −0.048; *p* = 0.004**), together with higher variant type allele frequencies for SNP6 (Estim = +0.043; *p* = 0.007**) were denoted as distinctive of the ssp. *ligustica* genotype based on the significance of the statistical model. For ssp. *mellifera*, the significant model assigned lower variant type allele frequencies for SNP1 and SNP6 as characteristic of this subspecies (Estim = −0.054; *p* = 0.001*** and Estim = −0.047; *p* = 0.002**, respectively).

### 3.5. Phylogenetic Lineage-Specific Logistic Regression Models

Driven by the above results and similar trends between phylogenetically related subspecies, we constructed a logistic regression model with the genetic bee lineage as the dummy-coded variable (C-lineage = ‘1’, M-lineage = ‘0’) and the percentages of variant type allele for each of the eight SNPs as continuous predictor variables (Table 1). The significant model depicted higher percentages of the variant type allele for SNP1 and SNP6 as being characteristic of subspecies of the C-lineage (Estim = +0.065; *p* < 0.001*** and Estim = +0.056; *p* = 0.001***, respectively), a finding that could be observed in the significance matrices of the pairwise Dunn’s Tests as well. Notably, although similar distributions between phylogenetically related subspecies were observed for SNP8, this SNP was not a significant predictor of the dummy-coded genetic lineage, probably due to the marginal significance of differences between samples of the ssp. *Mellifera* and the ssp. *carnica* genotypes (cf. Figure 4).

## 4. Discussion

Through additional information on the subspecies genotype of some sampled colonies, our results show that the variant type allele frequencies of the eight DBR-associated genetic variants in pooled worker bee samples are correlated with the *A. mellifera* subspecies and phylogenetic lineage and that this is reflected in the country-wise comparisons.

For example, samples of the ssp. *iberiensis* showed remarkable differences in variant type allele frequencies for many SNPs (i.e., SNP1, SNP2, SNP6 and SNP8) when compared to other subspecies. These differences are also mirrored in the variant type allele frequency distributions of the Portuguese samples, the only country representing ssp. *iberiensis*. Samples genotyped as ssp. *ligustica* showed variant type allele frequencies for SNP6, SNP7 and SNP8 that differed significantly from other subspecies, a finding that is reflected in the post-hoc pairwise tests comparing samples from Italy with other countries too. Countries with samples from multiple subspecies belonging to the same phylogenetic lineage, for example Romania with ssp. *ligustica*, ssp. *carpatica* and ssp. *carnica* samples (all C lineage), did not show highly dispersed allele frequencies when grouped country-wise. On the contrary, when focussing on countries including samples of multiple subspecies with distinct phylogenetic backgrounds, for example the Netherlands and Switzerland with ssp. *mellifera* (M lineage) and ssp. *carnica* (C lineage) (cf. Figure 3), high variance in variant type allele frequencies could be seen for most SNPs.

The strongest associations between subspecies genotype and variant type allele frequency distributions were found for SNP1, SNP2, SNP6 and SNP8. When considering the main subspecies in the two previously described modelling studies, i.e., ssp. *mellifera* -evolved in the Amsterdam Water Dune population (T. Blacquière, personal communication) and ssp. *carnica* from the population-wide modelling in Flanders (Belgium) [36], significant differences in variant type allele frequency distributions between ‘EUR_mel’ and ‘EUR_car’ were found for SNP1 (****), SNP2 (**), SNP5 (*), SNP6 (**) and SNP8 (*). In the population-wide study of ssp. *carnica* samples in Flanders, a reduced three-variant model with SNP2, SNP4 and SNP6 was obtained, in which SNP2 switched property from being a risk mutation in the original Amsterdam Water Dune model to being a protective mutation in the new study, and SNP6 switched from originally being protective to being a risk mutation in the population-wide ssp. *carnica* study. Aside from these results, SNP4 remained a risk mutation in both the original eight-variant model (Amsterdam Water Dune) and the new reduced three-variant model (ssp. *carnica*).

Altogether, the abovementioned findings suggest that the property of a DBR-associated SNP, meaning the SNP is either a risk or protective mutation relative to varroa reproduction, may vary with the variant type allele frequency of that SNP and thus the specific subspecies/genetic lineage the genotype–phenotype model is constructed from. More specifically, these results seem to underpin our hypothesis that, as genetic markers are linked to loci in genes that control the trait of interest, DBR marker–loci linkages may differ between *A. mellifera* subspecies or phylogenetic lineages. These differences in linkages would then be reflected in the properties of the described DBR-associated SNPs.

Except for SNP7 between ssp. *carnica* and ssp. *ligustica* samples, no significant differences in variant type allele frequencies were detected between subspecies belonging to the same genetic lineage (C or M). Therefore, it is possible that population-wide modelling studies considering subspecies belonging to the C lineage (other than ssp. *carnica*) will result in (more or less) the same SNP properties as in the recently published study for ssp. *carnica* in Flanders (Belgium), although this should be validated rigorously [36]. Moreover, as the original eight variants were derived from only a single ssp. *mellifera*-evolved colony, similar population-wide modelling studies should be performed for ssp. *mellifera* and ssp. *iberiensis*. Both of these subspecies should be considered separately since recent studies categorize ssp. *iberiensis* in the A lineage rather than the M lineage, especially when considering colonies from southern Spain and Portugal [41,48,49].

It is important to note that the above-stated hypothesis of marker–loci linkage dissimilarity between different subspecies or genetic lineages can only be validated by characterization of the loci residing in the trait-related genes. These DBR-related loci are currently unknown and identifying them would enable us to determine the genotypes of both the markers and the loci, thereby testing the linkage dissimilarity hypothesis.

The constructed logistic regression models showed that percentages of variant type alleles for SNP1 *and* SNP6 are remarkably predictive for sample classification into the phylogenetical C- or M-lineage (cf. Table 1). More specifically, if the percentages of variant type alleles for SNP1 *and* SNP6 in a pooled worker bee sample are known, one could calculate the probability of the pooled bees belonging to one of the two lineages. For example, if the pooled bee sample only contains variant type alleles for SNP1 *and* SNP6 (thus 100% Vt for both), the probability of the bees belonging to the C-lineage, according to the model, is 100%. On the contrary, this probability drops to only 2.5% if the sample does not contain any variant type allele for both SNPs (thus 0% Vt). Of course, these probabilities only count for the pooled samples themselves, they do not give any information on the individual bees in the samples.

In line with the two previously described modelling studies considering the eight DBR-associated markers, our results show that determining local honey bee subspecies genotypes before implementing marker-assisted selection for suppression of mite reproduction is of utmost importance. However, only one model has yet been validated on a population-wide scale for a specific subspecies and region, namely the three variants associated with DBR in ssp. *carnica* in Flanders, Belgium [36]. A future objective evolving from the current study could be the population-wide testing of the eight DBR-associated genetic variants in many *A. mellifera* subspecies across the European continent and evaluating the properties of the eight genetic markers by comparing them between different subspecies or regions. Such studies could follow the same experimental setup and protocols as described in [36], and are relatively inexpensive to perform.

It is hard to predict to what extent marker-assisted selection (MAS) based on (a subset of) the eight genetic variants associated with DBR will influence holistic colony-level mite resistance (i.e., the combination of varroa-resistance traits, including mite non-reproduction, varroa-sensitive hygiene, grooming behavior, ...). Therefore, when being implemented in selection programs for resilience against *V. destructor*, it will be important to thoroughly consider and evaluate colonies’ performances on these traits, as well as on other traits such as honey production, gentleness, behavior, etc. Only after validation in the considered population may these genetic markers be implemented in local MAS programs focusing on honey bee resilience against the varroa mite.

## Figures and Tables

**Table 1:** Logistic regression models with dummy-coded subspecies or genetic lineage (C vs. M) as output variable and the percentages of variant type alleles for each of the eight SNPs as continuous predictor variables. Significant regression models could only be constructed for ssp. *carnica*, ssp. *ligustica* and ssp. *mellifera* as a dummy-coded outcome, and for C- vs. M-lineage as a dummy-coded outcome. N_1_ = number of true cases belonging to the represented subspecies or the C-lineage; N_0_ = number of true cases belonging to “another” subspecies (but not known which one) or the M-lineage. In significant models, only significant SNPs and the intercept are represented. Estim.: estimate of the predictor (i.e., change in Log Odds for belonging to the represented subspecies/C-lineage (dummy-coded as 1) with every 1% increase in variant type allele frequency for the significant SNP); Sig.: significance of the predictor/intercept; NPV: Negative Predictive Value of the model; PPV: Positive Predictive Value of the model; Sens.: sensitivity of the model; Spec.: specificity of the model. Significance levels: *p* ≤ 0.05*; *p* ≤ 0.01**; *p* ≤ 0.001***. Significance level α was set at 0.05.

Subspecies	Observed Cases	SNP	Estim	Sig.	NPV	PPV	Sens.	Spec.
Adami	N_1_ = 2 N_0_ = 128	No significant model						
**Carnica**	N_1_ = 59 N_0_ = 71	**SNP1**	**0.059**	<0.001 ***	0.75	0.74	0.68	0.80
		Intercept	−3.997	0.004 **				
Carpatica	N_1_ = 4 N_0_ = 126	No significant model						
Iberiensis	N_1_ = 7 N_0_ = 123	No significant model						
**Ligustica**	N_1_ = 23 N_0_ = 107	**SNP1**	**−0.043**	0.044 *	0.93	0.83	0.65	0.97
		**SNP6**	**0.043**	0.007 **				
		**SNP8**	**−0.048**	0.004 **				
		Intercept	−3.323	0.136				
**Mellifera**	N_1_ = 23 N_0_ = 107	**SNP1**	**−0.054**	0.001 ***	0.86	0.66	0.60	0.88
		**SNP6**	**−0.047**	0.002 **				
		Intercept	1.909	0.228				
**Lineage**	**Observed cases**	**SNP**	**Estim**	**Sig.**	**NPV**	**PPV**	**Sens.**	**Spec.**
**C vs. M**	N_1_ = C = 88 N_0_ = M = 42	**SNP1**	**0.065**	<0.001 ***	0.86	0.89	0.94	0.76
		**SNP6**	**0.056**	0.001 ***				
		Intercept	−3.657	0.077				

## Data Availability

Dataset available on request from the authors.

## Supplementary Materials

The following supporting information can be downloaded at: https://www.mdpi.com/article/10.3390/insects15060419/s1, Table S1: Coordinates of apiary sites included in Tier 1 of the EU-funded B-GOOD project; Table S2: Genomic locations of the eight genetic variants associated with DBR in the hybrid DBBB Amsterdam Water Dune colony; Table S3: Summary of variant type allele frequency distributions of the eight genetic variants associated with DBR in pooled worker bee samples from colonies across the European Continent; Table S4: Summary of variant type allele frequency distributions of the eight genetic variants associated with DBR in pooled worker bee samples from different subspecies.
