# Supplementary material for: Allele Frequencies of Genetic Variants Associated with Varroa Drone Brood Resistance (DBR) in Apis mellifera Subspecies across the European Continent
Source: Insects. 2024 Jun 4;15(6):419. doi: 10.3390/insects15060419 (PMC11203681; doi:10.3390/insects15060419)
Supplement: Supplementary file 1 [file insects-15-00419-s001.zip › Supplementary Materials.pdf]

Supplementary Materials

**Supplementary Table S1.** Coordinates of apiary sites included in Tier 1 of the EU-funded B-GOOD project. This project comprised a three-tiered sampling structure of honey bee hives: Tier 1 hives were sampled for three consecutive years, Tier 2 hives were sampled in the project's latter two years and hives in Tier 3 were sampled in the last year only. Tier 1 included apiaries of eight partner institutions located in Germany (DE), the United Kingdom (UK), Belgium (BE), the Netherlands (NL), Romania (RO), France (FR), Switzerland (CH) and Portugal (PT). The coordinates (latitude and longitude) of the apiaries of these partner institutions are shown below. In Tier 2, five partners (from DE, NL, CH, IT and FI) each guided eight local beekeepers in the monitoring of three of their colonies. Due to privacy reasons, the B-GOOD project promised confidentiality for the coordinates of the apiaries of these beekeepers (indicated as NA). Tier 3 included 58 beekeepers on a Pan-European scale. Due to the same privacy reasons and confidentiality as mentioned for Tier 2, we are unable to report the coordinates of the sampling sites from beekeepers involved in Tier 3 (*cf.* NA). Tier3 included beekeepers from Germany (DE), Belgium (BE), the Netherlands (NL), France (FR), Switzerland (CH), Portugal (PT), Sweden (SE), Greece (GR), Italy (IT), Latvia (LV) and Poland (PL).				
**Sampling Tier**	**Included countries**	**Apiary site coordinates**		**Map**
		**Latitude (°N)**	**Longitude (°E)**	
Tier 1	DE	51.505	11.942	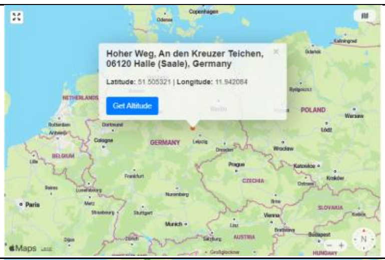
	UK	52.948	-1.068	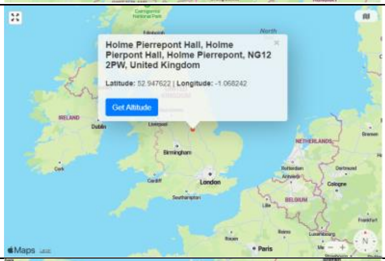
	BE	51.074	3.576	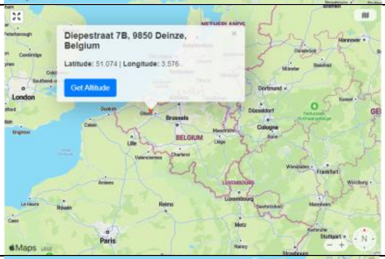
	NL	51.655	5.637	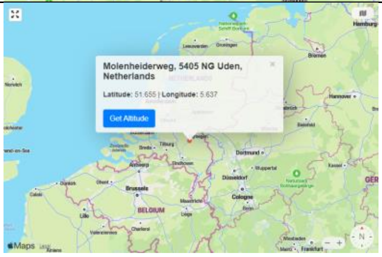

	RO	46.760	23.574	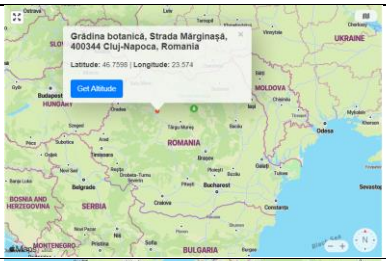
	FR	43.917	4.884	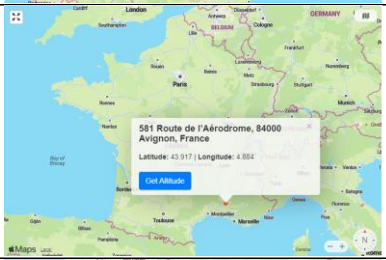
	CH	46.967	7.397	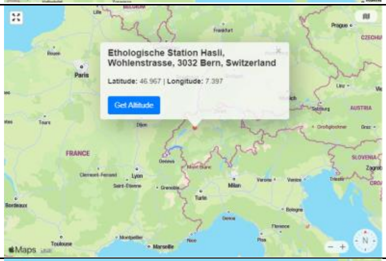
	PT	41.155	-7.694	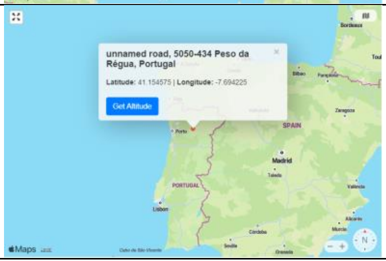
Tier 2	DE, NL, CH, IT, FI	NA	NA	NA
Tier 3	DE, BE, NL, FR, CH, PT, SE, GR, IT, LV, PL	NA	NA	NA

Supplementary Table S2. Genomic locations of the eight genetic variants associated with DBR in the hybrid DBBB Amsterdam Water Dune colony. Linkage groups (LG), positions and gene symbols are valid for reference genome Amel_HAv3.1. SNP numbers are allocated as in [1]. The last column describes the wild-type (Wt) to variant -type (Vt) allele conversion on genomic DNA level.

SNP	LG	Position	Gene symbol	Gene	Variant
1	LG1	24214694	LOC412088	Mucin-12 isoform X1	C>T
2	LG1	24214744	LOC412088	Mucin-12 isoform X1	T>C
3	LG3	11807235	LOC724886	Uncharacterized protein LOC724886 isoform X2	G>A
4	LG9	11542136	LOC100578770	Uncharacterized protein LOC100578770	T>C
5	LG9	11461121	LOC411744	Spectrin beta chain isoform X1	A>C
6	LG10	6509050	LOC408302	Solute carrier family 22 member 21	C>T
7	LG15	4695209	LOC410626	Sodium-coupled monocarboxylate transporter 1	C>T
8	LG15	5701853	LOC551562	Dynein beta chain, ciliary	T>C

Supplementary Table S3. Summary of variant type allele frequency distributions of the eight genetic variants associated with DBR in pooled worker bee samples from colonies across the European continent.

		%Vt SNP1	%Vt SNP2	%Vt SNP3	%Vt SNP4	%Vt SNP5	%Vt SNP6	%Vt SNP7	%Vt SNP8
Belgium	Mean	63,86	62,90	38,64	55,05	53,49	45,48	4,24	37,67
	Std. Error of Mean	7,409	6,485	4,635	3,735	2,242	4,140	2,120	4,807
	Variance	1207,8	883,1	472,6	306,9	110,6	377,1	98,8	508,3
	Std. Dev.	34,753	29,718	21,738	17,518	10,517	19,420	9,942	22,545
	Median	71,22	70,19	44,35	56,56	53,61	41,96	,00	41,12
	N	22,0	21,0	22,0	22,0	22,0	22,0	22,0	22,0
	Minimum	0,7	0	0	19,9	29,5	14,5	0	1,0
	Maximum	100,0	100,0	73,3	87,7	72,9	79,6	30,3	71,4
Finland	Mean	42,43	57,74	46,82	53,77	51,76	59,82	5,62	36,42
	Std. Error of Mean	6,395	6,512	4,539	4,195	2,495	7,227	1,874	6,957
	Variance	817,9	848,1	412,0	351,9	124,5	940,2	70,2	967,9
	Std. Dev.	28,600	29,123	20,298	18,759	11,160	30,662	8,379	31,111
	Median	42,72	68,93	46,96	59,32	46,99	72,60	,00	37,38
	N	20,0	20,0	20,0	20,0	20,0	18,0	20,0	20,0
	Minimum	3,1	0	0	2,1	39,2	0	0	0
	Maximum	89,1	99,7	85,7	91,3	74,7	100,0	22,6	100,0
France	Mean	31,64	47,59	45,33	49,84	30,67	32,04	9,26	55,85
	Std. Error of Mean	6,689	8,249	6,941	5,629	5,350	5,484	4,508	5,157
	Variance	626,3	952,6	674,5	443,6	372,1	421,0	284,5	345,8
	Std. Dev.	25,026	30,864	25,971	21,062	19,289	20,518	16,867	18,595
	Median	26,57	49,61	48,00	46,98	34,07	30,97	,00	64,43
	N	14,0	14,0	14,0	14,0	13,0	14,0	14,0	13,0
	Minimum	7,0	3,4	0	9,8	0,4	1,1	0	0
	Maximum	92,5	96,5	89,3	87,8	66,9	77,6	56,8	68,9
Germany	Mean	63,80	63,33	43,93	56,85	47,82	51,27	3,97	38,22
	Std. Error of Mean	3,036	2,394	2,266	2,790	1,677	2,817	1,240	2,617
	Variance	589,8	372,5	333,7	505,9	182,9	515,9	99,9	445,2
	Std. Dev.	24,285	19,301	18,266	22,491	13,524	22,714	9,995	21,099
	Median	66,09	63,31	46,23	58,99	49,10	45,91	,00	44,68
	N	64,0	65,0	65,0	65,0	65,0	65,0	65,0	65,0
	Minimum	13,4	13,8	0	3,7	2,6	15,3	0	0
	Maximum	100,0	97,9	80,2	100,0	89,2	100,0	49,5	65,6
Greece	Mean	75,48	84,08	50,41	46,64	54,89	68,15	2,06	52,96
	Std. Error of Mean	7,633	1,631	6,567	6,714	6,336	1,305	2,056	1,120
	Variance	174,8	8,0	129,4	135,3	120,4	3,4	12,7	3,8
	Std. Dev.	13,221	2,825	11,375	11,630	10,975	1,846	3,561	1,941
	Median	76,48	84,35	52,52	40,19	50,96	68,15	0	53,94
	N	3,0	3,0	3,0	3,0	3,0	2,0	3,0	3,0
	Minimum	61,8	81,1	38,1	39,7	46,4	66,8	0	50,7
	Maximum	88,2	86,8	60,6	60,1	67,3	69,5	6,2	54,2
Italy	Mean	57,26	77,69	34,48	63,49	46,79	81,09	13,56	16,36
	Std. Error of Mean	4,804	3,692	4,677	2,876	2,433	2,880	2,563	3,327
	Variance	715,3	422,7	678,2	256,4	183,5	240,5	203,7	343,1
	Std. Dev.	26,745	20,559	26,042	16,012	13,546	15,509	14,272	18,524
	Median	60,31	84,85	34,37	66,74	48,51	84,49	7,50	3,27

	N	31,0	31,0	31,0	31,0	31,0	29,0	31,0	31,0
	Minimum	9,5	28,8	0	24,2	14,1	45,3	0	0
	Maximum	98,4	100,0	80,5	88,8	67,9	100,0	42,3	53,6
Latvia	Mean	26,52	31,84	46,76	52,44	41,12	41,13	7,33	40,94
	Std. Error of Mean	8,709	12,242	13,200	7,660	7,418	4,740	5,506	5,665
	Variance	303,4	599,5	697,0	234,7	220,1	112,3	121,3	128,4
	Std. Dev.	17,418	24,485	26,401	15,320	14,837	10,599	11,013	11,330
	Median	25,44	31,35	54,65	58,56	41,63	39,19	3,02	44,00
	N	4,0	4,0	4,0	4,0	4,0	5,0	4,0	4,0
	Minimum	6,5	7,7	9,1	29,7	25,2	26,2	0	25,6
	Maximum	48,7	57,0	68,7	62,9	56,0	52,1	23,3	50,1
Poland	Mean	48,39	42,01	41,84	33,26	35,00	30,71	3,49	69,68
	Std. Error of Mean	9,204	6,646	5,341	3,028	3,885	6,883	1,464	4,641
	Variance	1524,8	795,1	513,5	165,0	271,7	852,7	38,6	387,7
	Std. Dev.	39,049	28,198	22,660	12,845	16,484	29,201	6,210	19,691
	Median	44,91	53,96	51,09	34,25	39,72	28,77	,00	67,41
	N	18,0	18,0	18,0	18,0	18,0	18,0	18,0	18,0
	Minimum	5,7	0	0	13,1	0	0	0	36,0
	Maximum	100,0	76,3	75,4	69,1	55,5	100,0	21,9	100,0
Portugal	Mean	2,43	,39	45,84	32,42	43,16	0,04	0	98,61
	Std. Error of Mean	0,164	,390	5,986	4,344	4,921	0,040	0	1,389
	Variance	0,3	1,8	429,9	226,5	290,6	0	0	23,2
	Std. Dev.	0,570	1,349	20,735	15,049	17,046	0,139	0	4,813
	Median	2,31	,00	50,70	35,27	47,18	0	0	100,00
	N	12,0	12,0	12,0	12,0	12,0	12,0	12,0	12,0
	Minimum	1,7	0	0	9,4	7,1	0	0	83,3
	Maximum	3,4	4,7	72,0	50,6	63,9	0,5	0	100,0
Romania	Mean	87,44	83,61	33,38	50,90	45,23	51,64	0,01	40,09
	Std. Error of Mean	3,326	2,408	7,633	8,033	4,932	3,795	0,009	4,422
	Variance	88,5	46,4	466,1	516,2	194,6	115,2	,0	156,4
	Std. Dev.	9,409	6,812	21,588	22,720	13,948	10,733	,026	12,507
	Median	89,70	82,59	31,12	54,83	42,84	51,82	,00	44,76
	N	8,0	8,0	8,0	8,0	8,0	8,0	8,0	8,0
	Minimum	67,8	74,0	0	8,7	29,8	38,6	0	17,4
	Maximum	100,0	93,1	69,6	80,7	72,5	73,3	0,1	56,1
Sweden	Mean	35,68	48,92	50,78	62,82	37,77	36,18	18,84	49,40
	Std. Error of Mean	4,516	5,408	5,687	6,421	5,581	3,700	4,665	4,187
	Variance	326,3	438,8	485,2	659,7	467,2	219,0	348,2	262,9
	Std. Dev.	18,064	20,947	22,026	25,685	21,614	14,800	18,661	16,214
	Median	35,07	48,48	56,48	62,76	47,24	33,88	14,31	48,85
	N	16,0	15,0	15,0	16,0	15,0	16,0	16,0	15,0
	Minimum	4,5	13,4	4,6	16,9	0	17,5	0	0
	Maximum	62,7	87,6	82,6	100,0	61,1	62,2	48,7	65,2
Switzer-land	Mean	60,85	50,31	43,11	62,71	43,73	47,12	3,74	49,68
	Std. Error of Mean	4,310	3,576	3,385	3,747	1,724	4,717	1,545	2,897
	Variance	761,6	524,4	469,7	575,8	121,9	912,1	97,9	344,1
	Std. Dev.	27,598	22,900	21,673	23,995	11,042	30,201	9,893	18,551
	Median	64,07	48,00	46,26	66,27	43,95	43,35	0	52,93
	N	41,0	41,0	41,0	41,0	41,0	41,0	41,0	41,0
	Minimum	14,8	0	0	20,9	4,9	1,2	0	0

	Maximum	100,0	89,3	83,9	100,0	60,2	100,0	40,0	76,4
The Nether-lands	Mean	37,54	50,06	39,55	54,60	48,18	29,75	8,18	49,76
	Std. Error of Mean	2,616	2,562	2,235	2,402	2,379	1,907	1,418	1,777
	Variance	663,6	636,6	479,4	553,7	532,0	349,2	193,1	299,8
	Std. Dev.	25,760	25,232	21,895	23,531	23,065	18,688	13,896	17,315
	Median	32,02	47,50	44,04	54,52	47,48	30,45	,00	52,52
	N	97,0	97,0	96,0	96,0	94,0	96,0	96,0	95,0
	Minimum	0	0	0	0	6,1	0	0	0
	Maximum	100,0	100,0	81,3	100,0	100,0	78,9	58,8	79,9
United Kingdom	Mean	22,27	45,28	33,17	45,55	33,53	32,96	14,20	55,10
	Std. Error of Mean	5,535	9,667	6,817	5,601	5,950	10,246	5,401	5,366
	Variance	214,4	654,1	325,3	219,6	247,8	839,8	204,2	201,5
	Std. Dev.	14,644	25,576	18,036	14,819	15,742	28,980	14,291	14,196
	Median	18,59	48,14	28,82	45,44	39,08	28,11	15,45	60,03
	N	7,0	7,0	7,0	7,0	7,0	8,0	7,0	7,0
	Minimum	7,6	11,1	14,4	29,9	0	0	0	29,3
	Maximum	50,0	76,3	70,0	71,7	48,8	78,0	33,0	66,0
Total	Mean	48,60	54,62	41,61	54,70	45,70	42,79	6,87	45,89
	Std. Error of Mean	1,612	1,455	1,148	1,182	,938	1,451	,664	1,295
	Variance	927,4	754,1	469,2	498,8	310,6	744,9	157,3	593,3
	Std. Dev.	30,453	27,461	21,662	22,334	17,625	27,294	12,544	24,358
	Median	48,42	57,62	45,97	54,61	46,67	39,86	,00	49,60
	N	357,0	356,0	356,0	357,0	353,0	354,0	357,0	354,0
	Minimum	0	0	0	0	0	0	0	0
	Maximum	100,0	100,0	89,3	100,0	100,0	100,0	58,8	100,0

Supplementary Table S4. Summary of variant type allele frequency distributions of the eight genetic variants associated with DBR in pooled worker bee samples from different subspecies.

		%Vt SNP1	%Vt SNP2	%Vt SNP3	%Vt SNP4	%Vt SNP5	%Vt SNP6	%Vt SNP7	%Vt SNP8
BE_car	Mean	80,4150	74,8375	37,4325	62,3725	60,9388	53,5875	3,9363	35,9225
	Std. Error of Mean	7,91173	6,88273	6,07270	5,81795	3,01127	8,19228	3,76165	8,06359
	Variance	500,764	378,976	295,022	270,788	72,542	536,907	113,200	520,172
	Std. Dev.	22,37775	19,46731	17,17620	16,45564	8,51716	23,17126	10,63954	22,80729
	Median	88,1600	74,6100	41,1450	64,2500	61,5650	59,2800	0	46,4200
	N	8	8	8	8	8	8	8	8
	Minimum	45,40	48,11	0	26,45	47,32	14,53	0	0,98
	Maximum	100,00	100,00	53,62	78,58	72,88	79,59	30,25	60,08
CH_car	Mean	77,1050	60,2417	52,2567	78,8600	45,8692	53,2900	7,2525	52,7800
	Std. Error of Mean	8,12291	5,65876	4,54918	4,71400	3,11943	8,69125	4,20528	4,46980
	Variance	791,781	384,259	248,340	266,662	116,770	906,453	212,212	239,749
	Std. Dev.	28,13860	19,60252	15,75882	16,32979	10,80602	30,10736	14,56750	15,48383
	Median	85,0300	57,3800	53,5650	75,5550	47,6050	49,6450	,0000	54,9300
	N	12	12	12	12	12	12	12	12
	Minimum	14,84	33,93	22,27	51,34	26,53	11,04	0	22,80
	Maximum	100,00	89,27	80,68	100,00	59,34	100,00	40,00	71,78
CH_mel	Mean	35,2850	40,7600	32,8167	55,8500	40,4950	17,1233	0	54,5683
	Std. Error of Mean	6,37832	6,31737	9,99834	11,08777	7,80641	5,66874	0	11,30133
	Variance	244,098	239,455	599,801	737,632	365,640	192,808	0	766,320
	Std. Dev.	15,62363	15,47433	24,49084	27,15937	19,12173	13,88553	0	27,68248
	Median	28,5900	36,9850	38,4150	70,6350	44,4100	10,7400	0	63,0650
	N	6	6	6	6	6	6	6	6
	Minimum	20,94	22,09	0	20,90	4,89	4,32	0	0
	Maximum	63,57	67,88	63,57	76,29	60,18	40,40	0	76,37
DE_car	Mean	59,0329	61,7480	42,6200	53,2944	49,3400	52,1488	2,7668	36,2216
	Std. Error of Mean	5,11881	4,37640	3,75055	4,93991	3,32787	4,37990	1,43166	4,22722
	Variance	628,852	478,822	351,666	610,067	276,868	479,588	51,241	446,735
	Std. Dev.	25,07692	21,88200	18,75277	24,69953	16,63936	21,89950	7,15828	21,13610
	Median	59,4050	60,7300	41,1600	51,1800	48,5000	45,1500	,0000	44,6800
	N	24	25	25	25	25	25	25	25
	Minimum	13,38	14,32	0	7,79	23,51	19,52	0	0
	Maximum	100,00	93,82	80,17	100,00	89,18	99,42	29,96	65,62
EUR_adam	Mean	40,8800	52,4350	52,7550	44,2800	56,7500	53,4150	0	32,2800
	Std. Error of Mean	18,78000	21,87500	8,03500	17,62000	10,19000	27,03500	0	29,85000
	Variance	705,377	957,031	129,122	620,929	207,672	1461,782	0	1782,045
	Std. Dev.	26,55893	30,93592	11,36321	24,91844	14,41084	38,23326	0	42,21427
	Median	40,8800	52,4350	52,7550	44,2800	56,7500	53,4150	0	32,2800
	N	2	2	2	2	2	2	2	2
	Minimum	22,10	30,56	44,72	26,66	46,56	26,38	0	2,43
	Maximum	59,66	74,31	60,79	61,90	66,94	80,45	0	62,13
EUR_car	Mean	68,6630	64,4692	44,1161	59,6740	51,9687	52,0677	3,6992	39,3266
	Std. Error of Mean	3,14806	2,85042	2,28731	3,05589	2,18402	2,89105	1,14506	2,60014
	Variance	604,529	503,743	324,370	578,983	290,966	518,207	81,293	412,404
	Std. Dev.	24,58717	22,44422	18,01029	24,06207	17,05773	22,76416	9,01625	20,30773
	Median	72,8300	66,4150	45,1450	62,8350	49,4200	48,2750	0	46,7200
	N	61	62	62	62	61	62	62	61

	Minimum	13,38	1,36	0	7,79	23,51	11,04	0	,00
	Maximum	100,00	100,00	80,68	100,00	100,00	100,00	40,00	71,78
EUR_carp	Mean	66,6500	65,1200	30,8525	65,0475	45,9050	37,9575	0	51,5775
	Std. Error of Mean	21,02606	21,00510	14,95467	7,89606	9,34543	12,64669	0	3,83470
	Variance	1768,381	1764,856	894,568	249,391	349,348	639,755	0	58,820
	Std. Dev.	42,05212	42,01019	29,90934	15,79212	18,69085	25,29339	0	7,66940
	Median	79,8150	82,0100	26,9150	68,0700	40,6550	47,2600	0	50,8900
	N	4	4	4	4	4	4	4	4
	Minimum	7,00	3,37	0	43,38	29,78	1,14	0	44,51
	Maximum	99,97	93,09	69,58	80,67	72,53	56,17	0	60,02
EUR_iber	Mean	2,1557	0,6671	50,6329	31,7714	38,1943	0,0686	0	97,6186
	Std. Error of Mean	0,16395	0,66714	7,57888	5,21364	7,80201	0,06857	0	2,38143
	Variance	0,188	3,116	402,076	190,274	426,100	0,033	0	39,698
	Std. Dev.	0,43378	1,76509	20,05183	13,79398	20,64219	0,18142	0	6,30067
	Median	2,1200	0	55,0700	30,4100	42,5900	0	0	100,0000
	N	7	7	7	7	7	7	7	7
	Minimum	1,72	0	16,81	13,67	7,08	0	0	83,33
	Maximum	2,94	4,67	72,04	48,51	63,94	0,48	0	100,00
EUR_lig	Mean	52,4792	72,7715	27,5946	60,8715	46,6088	75,5257	11,2223	18,9554
	Std. Error of Mean	5,61016	4,86971	4,26887	3,27055	3,05896	3,81352	2,67183	4,44451
	Variance	818,322	616,566	473,806	278,109	243,288	334,488	185,605	513,596
	Std. Dev.	28,60632	24,83075	21,76708	16,67660	15,59769	18,28901	13,62371	22,66266
	Median	50,1300	81,9100	25,7250	60,8300	47,3600	79,5300	3,4700	1,5600
	N	26	26	26	26	26	23	26	26
	Minimum	9,48	12,95	0	31,52	2,63	25,80	0	0
	Maximum	98,38	100,00	66,79	88,80	73,85	100,00	38,52	62,69
EUR_mel	Mean	25,0914	38,5231	41,1057	49,8806	37,0074	25,2983	10,6186	57,4086
	Std. Error of Mean	3,81630	4,29297	3,71001	3,37033	3,28701	3,45909	2,82173	3,26221
	Variance	509,745	645,036	481,747	397,569	378,156	430,750	278,676	372,470
	Std. Dev.	22,57753	25,39757	21,94874	19,93912	19,44623	20,75452	16,69359	19,29949
	Median	19,9300	35,3000	39,9300	47,5500	39,0800	22,8650	0	63,3200
	N	35	35	35	35	35	36	35	35
	Minimum	0	0	0	18,48	0	0	0	0
	Maximum	92,54	96,51	85,70	100,00	100,00	77,96	57,81	100,00
FI_car	Mean	69,8950	82,7275	45,5875	68,9650	54,8175	73,5825	3,7825	36,1050
	Std. Error of Mean	9,56244	5,94878	1,22217	9,99885	4,01410	10,39759	1,95284	13,51289
	Variance	365,761	141,552	5,975	399,908	64,452	432,439	15,254	730,393
	Std. Dev.	19,12488	11,89756	2,44435	19,99771	8,02821	20,79518	3,90567	27,02579
	Median	70,7850	79,2950	45,7550	69,6200	54,1950	72,5950	3,1350	34,8200
	N	4	4	4	4	4	4	4	4
	Minimum	48,89	72,58	42,93	45,37	46,89	49,14	0	9,12
	Maximum	89,12	99,74	47,91	91,25	63,99	100,00	8,86	65,66
FI_lig	Mean	33,8300	50,5071	33,4771	52,1329	54,9086	77,7720	7,7729	27,2357
	Std. Error of Mean	10,56922	9,90497	8,49379	5,00583	4,99429	5,93050	3,50843	9,77464
	Variance	781,959	686,760	505,011	175,408	174,600	175,854	86,164	668,805
	Std. Dev.	27,96353	26,20610	22,47245	13,24417	13,21364	13,26099	9,28243	25,86127
	Median	18,5200	48,9400	46,2500	59,3700	47,6400	79,8900	2,2500	38,7200
	N	7	7	7	7	7	5	7	7
	Minimum	10,06	12,95	0	34,25	42,60	56,64	0	0
	Maximum	86,44	79,58	54,62	65,92	73,85	92,90	19,57	52,77

FI_mel	Mean	25,0125	24,9725	62,3825	45,6375	43,8175	10,2375	0	69,9850
	Std. Error of Mean	14,73638	14,85725	9,43959	4,38147	1,70489	5,86187	0	14,52275
	Variance	868,644	882,952	356,423	76,789	11,627	137,446	0	843,641
	Std. Dev.	29,47277	29,71451	18,87918	8,76294	3,40978	11,72374	0	29,04549
	Median	15,3900	20,5800	62,0550	47,7150	44,6750	7,3250	0	74,6700
	N	4	4	4	4	4	4	4	4
	Minimum	3,13	0	39,72	34,22	39,25	0	0	30,60
	Maximum	66,14	58,73	85,70	52,90	46,67	26,30	0	100,00
FR_mel	Mean	50,9133	61,2067	49,5267	62,0467	27,3467	20,3800	15,5667	64,8900
	Std. Error of Mean	23,34410	27,56689	13,20491	6,73591	7,86472	11,08365	7,82460	0,08737
	Variance	1634,841	2279,800	523,109	136,118	185,562	368,542	183,673	0,023
	Std. Dev.	40,43316	47,74725	22,87157	11,66695	13,62210	19,19744	13,55260	0,15133
	Median	48,4100	80,2300	48,3800	56,5200	34,0700	14,6500	21,9600	64,9400
	N	3	3	3	3	3	3	3	3
	Minimum	11,79	6,88	27,25	54,17	11,67	4,70	0	64,72
	Maximum	92,54	96,51	72,95	75,45	36,30	41,79	24,74	65,01
IT_lig	Mean	58,3506	81,6353	24,2894	64,4606	45,1600	79,2506	13,9588	11,4406
	Std. Error of Mean	6,41749	4,72378	5,24182	4,18221	3,01472	3,81335	3,66935	4,19889
	Variance	700,131	379,339	467,104	297,345	154,506	232,666	228,890	299,722
	Std. Dev.	26,45999	19,47664	21,61260	17,24370	12,43003	15,25340	15,12912	17,31247
	Median	54,9200	89,1400	25,0200	63,2700	44,0300	82,0100	4,8500	0
	N	17	17	17	17	17	16	17	17
	Minimum	9,48	28,78	0	31,52	17,95	45,29	0	0
	Maximum	98,38	100,00	66,79	88,80	63,84	100,00	38,52	45,98
NL_car	Mean	63,8378	53,1878	52,4789	52,8178	64,2688	39,3278	2,9478	37,3438
	Std. Error of Mean	6,09055	9,95243	5,35205	8,37827	10,13915	3,80888	2,11817	6,34678
	Variance	333,853	891,458	257,800	631,759	822,420	130,568	40,380	322,253
	Std. Dev.	18,27165	29,85730	16,05615	25,13481	28,67786	11,42664	6,35450	17,95141
	Median	57,9700	56,1200	51,6400	56,1800	54,2950	39,6200	0	39,8650
	N	9	9	9	9	8	9	9	8
	Minimum	42,57	1,36	19,77	13,28	31,56	14,89	0	0
	Maximum	100,00	100,00	74,33	100,00	100,00	52,04	19,02	54,34
NL_mel	Mean	17,1853	33,5493	40,7660	48,2113	37,3507	29,4847	15,0367	54,7727
	Std. Error of Mean	5,03877	5,60675	5,53578	5,81704	6,36633	4,86516	5,47665	4,41949
	Variance	380,838	471,535	459,673	507,570	607,953	355,046	449,905	292,979
	Std. Dev.	19,51507	21,71485	21,43999	22,52931	24,65669	18,84267	21,21098	17,11662
	Median	12,0400	34,1700	51,4100	45,5800	38,2700	32,9200	1,4900	60,3900
	N	15	15	15	15	15	15	15	15
	Minimum	0	0,40	0	18,48	8,28	0	0	21,91
	Maximum	76,83	71,91	71,42	100,00	100,00	57,37	57,81	79,87
RO_car	Mean	87,2375	80,5475	22,1250	42,7275	41,3075	52,0050	0	32,3675
	Std. Error of Mean	1,90655	1,52865	7,57360	13,48987	3,03098	7,53007	0	6,61357
	Variance	14,540	9,347	229,438	727,906	36,747	226,808	0	174,957
	Std. Dev.	3,81309	3,05729	15,14720	26,97973	6,06197	15,06014	0	13,22714
	Median	88,0800	79,6250	27,1150	48,1150	42,8350	48,0600	0	31,5750
	N	4	4	4	4	4	4	4	4
	Minimum	81,90	78,02	0	8,66	32,74	38,61	0	17,42
	Maximum	90,89	84,92	34,27	66,02	46,82	73,29	0	48,90
UK_mel	Mean	22,2743	45,2857	33,1714	45,5514	33,5314	32,9550	14,2000	55,0986

	Std. Error of Mean	5,53465	9,66669	6,81609	5,60134	5,95005	10,24643	5,40148	5,36559
	Variance	214,427	654,115	325,214	219,625	247,822	839,915	204,232	201,527
	Std. Dev.	14,64332	25,57567	18,03368	14,81976	15,74235	28,98128	14,29097	14,19602
	Median	18,5900	48,1400	28,8200	45,4400	39,0800	28,1050	15,4500	60,0300
	N	7	7	7	7	7	8	7	7
	Minimum	7,56	11,12	14,44	29,91	0	0	0	29,34
	Maximum	49,97	76,29	70,02	71,75	48,83	77,96	33,03	66,01
Total	Mean	51,3332	57,1760	40,0618	56,4428	46,5062	47,1580	6,9474	41,7593
	Std. Error of Mean	1,98619	1,77634	1,30496	1,37196	1,14706	1,77695	0,79786	1,61797
	Variance	1005,963	810,937	437,649	483,745	335,516	798,863	163,601	667,549
	Std. Dev.	31,71692	28,47695	20,92006	21,99421	18,31711	28,26417	12,79066	25,83697
	Median	51,1100	57,7500	42,9300	59,2200	46,8500	45,1500	0	47,0000
	N	255	257	257	257	255	253	257	255
	Minimum	0	0	0	7,79	0	0	0	0
	Maximum	100,00	100,00	85,70	100,00	100,00	100,00	57,81	100,00

Reference

1. Bouuaert, D.C.; Van Poucke, M.; De Smet, L.; Verbeke, W.; de Graaf, D.C.; Peelman, L. qPCR assays with dual-labeled probes for genotyping honey bee variants associated with varroa resistance. *Bmc Vet. Res.* **2021**, *17*, 179.
